# Sympatric cleptobiotic stingless bees have species-specific cuticular profiles that resemble their hosts

**DOI:** 10.1038/s41598-022-06683-w

**Published:** 2022-02-16

**Authors:** Manuel Vázquez, David Muñoz, Rubén Medina, Robert J. Paxton, Favizia Freitas de Oliveira, José Javier G. Quezada-Euán

**Affiliations:** 1 Departamento de Apicultura Tropical, Campus de Ciencias Biológicas y Agropecuarias, Universidad Autónoma de Yucatán, Km 15.5 Carr., Xmatkuil, Mérida, Yucatán Mexico; 2 Facultad de Ingeniería Química, Campus de Ingenierías y Ciencias Exactas, Mérida, Yucatán Mexico; 3 Instituto Nacional de Investigaciones Forestales Agrícolas y Pecuarias (INIFAP), Campo Experimental Edzná, Campeche, Mexico; 4 Institute für Biology, Martin Luther University Halle-Wittenberg, 06099 Halle (Saale), Germany; 5 Laboratório de Bionomia, Biogeografia e Sistemática de Insetos (BIOSIS), Museu de História Natural da Bahia (MHNBA), Instituto de Biologia, Universidade Federal da Bahia, Rua Barão de Jeremoabo, Número 668, Campus de Ondina, Salvador, Bahia Brazil

**Keywords:** Community ecology, Ecology, Evolution

## Abstract

Stingless bees are the largest group of eusocial pollinators with diverse natural histories, including obligate cleptobionts (genus *Lestrimelitta*) that completely abandoned flower visitation to rely on other stingless bees for food and nest materials. Species of *Lestrimeliita* are thought to specialize upon different host species, and deception through chemical similarity has been proposed as a mechanism to explain this phenomenon. In the Yucatan Peninsula of Mexico, *Scaptotrigona pectoralis* is a species chemically distinct from, and not preferred as a host by, locally widespread *Lestrimeliita niitkib*; witnessing attacks on *S. pectoralis* colonies offered the opportunity to test the sensory deception hypothesis to cletoparasitism. Analysis of cuticular profiles revealed that the *Lestrimelitta* attacking *S. pectoralis* differed significantly in odour bouquet to *L. niitkib* and, in contrast, it resembled that of *S. pectoralis*. Further analyses, including morphometrics, mtDNA barcoding, and the examination of taxonomic features, confirmed the existence of two sympatric *Lestrimelitta* species. The results give support to the hypothesis of chemical deception as a cleptobiotic strategy in *Lestrimelitta* sp. This is the first evidence that sympatric cleptobionts of the same genus select hosts in accordance with species-specific cuticular profiles, with possible consequences for ecological adaptation and the evolution of these remarkable organisms and the community of stingless bee hosts.

## Introduction

Cleptobiosis is the behavior of stealing food, or sometimes nesting materials or other items of value, either from members of the same or a different species^1^. Given its obvious advantages, facultative cleptobiosis is widespread in the animal kingdom with most animal species occassionaly robbing food from conspecifics and heterospecifics. In contrast, obligate cleptobiosis is very rare, perhaps because it entails extreme specialization in a narrow niche and can only evolve under very specific circumstances^1^. Obligate highly eusocial cleptobionts have been found only in the Tribe Meliponini or stingless bees^2^.

The stingless bees are the most diversified group of highly eusocial bees encompassing over 500 species that are the predominant pollinators in the tropics worldwide^3^. One contrasting trait of stingless bees compared with the honey bee (Tribe Apini), the other group of highly eusocial bees, is its wide variation in morphology and lifestyles, sometimes with extreme adaptations^4^, among these the evolution of obligate cleptobiosis. Obligate cleptobiotic stingless bees are presently classified in two genera, the Paleotropical *Cleptotrigona* Moure, 1961, with one known species and the Neotropical *Lestrimelitta* Friese*,* 1903 with over two dozen species^5^. Cleptobiotic stingless bees represent a rare example of a highly specialized adaptation (including changes in morphology) as a consequence of having completely abandoned the collection of pollen, nectar and resins from plants to thrive exclusively on the robbing of resources and nest materials from other non-cleptiobiotic species of stingless bees^2^.

An interesting feature of cleptobiotic stingless bees is their preference for certain host stingless bee species to rob and, likewise, there are many potential host stingless bee species that are seldom or never raided. Moreover, different species seem to prey upon different hosts^2,6–9^. The mechanisms that *Lestrimelitta* use to overcome colony defenses and to select certain host species over others are poorly understood, although there are two basic strategies that cleptobionts can use to enter a target colony: force and chemical deception^2,10^. Because of its readily noticeable raids, it has been generally accepted that force and the use of citral (and other mandibular propaganda pheromones) may be the predominant mechanisms of host exploitation by *Lestrimelitta*^2,9^. However, recently, evidence for the possible role of chemical deception was obtained for *L. niitkib* of the Yucatan Peninsula^11^. Comparing the cuticular profiles of *L. niitkib* with its hosts has revealed similarity in the alkene fragment with some of *L. niitkib*’s preferred species (namely, *Nannotrigona perilampoides* Cresson*,* 1878 and *Plebeia* spp.) but a significantly distant profile with non-preferred ones like *Melipona (Melikerria) beecheii* Bennett, 1831 and *Scaptotrigona pectoralis* (Dalla Torre, 1896)^11^*.* Furthermore, potential host species with chemically distant profiles reacted more rapidly to the presence of *Lestrimelitta* workers in their colony (but not so chemically similar hosts), presumably because their different profiles allowed a fast detection of the intruder. This evidence suggested that sensory deception may be relevant in the selection and invasion of hosts by cleptobiotic stingless bees^11^.

Cuticular hydrocarbons (CHCs) are well known to play an important role in social insects^12^. Insect CHCs represent the dominant fraction of the waxy lipid layer located on insect epicuticle. The original role of CHCs is thought to be protection of the insect against desiccation^13^. However, CHCs secondarily evolved as important cues in chemical communication, most prominently in intra and inter specific recognition^14^. In stingless bees, unsaturated cuticular hydrocarbons, alkenes and alkadienes seem to be the main compounds responsible for nestmate recognition^15^. Because of their different ecological properties and specificity, CHCs have been proposed as candidate traits through which ecological adaptation could lead to selective mating and reproductive isolation^16–18^.

Long-term records of the incursion of *L. niitkib* on stingless bee yards have indicated that *S. pectoralis* is never attacked and that, on the contrary, cleptobiont colonies can be killed by the latter^7,19^. Given this background information, witnessing successful attacks of *Lestrimelitta* on *S. colonies* in the Eastern part of the Yucatan Peninsula was, thus, highly unusual and offered us the opportunity to test predictions of the hypothesis of sensory deception though chemical similarity. In addition, it allowed for a better understanding of the relative importance of chemical deception in *Lestrimelitta* ecology and, ultimately, to ask whether cuticular cues matter in the evolution of eusocial stingless bee cleptobionts and their hosts.

## Materials and methods

### Collection of biological material

Two separate attacks of *Lestrimelitta* sp*.* were witnessed on two different colonies of *S. pectoralis* kept in a stingless bee yard in the locality of Felipe Carrillo Puerto, Quintana Roo (Q Roo), Yucatan Península (19°35′02″ N 88°02′13″ W) (Supplementary files, Fig. S1 online). At each attack, two samples of arriving *Lestrimelitta* sp. workers were collected. One sample was used for chemical Gas Chromatography and Mass Spectrometry (GCMS) and consisted of the extracts from three individual workers that had their legs previously removed to avoid contamination with resin/pollen products, and that were sequentially submerged for 1 min in glass vials (Agilent Tech.) containing 500 µl of hexane. Another sample of at least ten workers was collected in Eppendorf tubes containing ethanol for morphometric analyses and DNA barcoding. In addition to the samples of attacking *Lestrimelitta*, we also collected the same type of samples and number of workers from five *S. pectoralis* colonies (including the two raided colonies) and from five *N. perilampoides* colonies (the preferred host species of *L. niitkib*) from QRoo. Host species and identity are well documented for *L. niitkib* in the state of Yucatan where attacks on *S. pectoralis* have not been witnessed^7,11^. As controls, we collected samples from Mérida in the neighboring state of Yucatan (Supplementary files, Fig. S1 online), from five colonies of the two same putative host species using similar procedures and number of individuals. Samples from five colonies of *L. niitkib* were also collected in Yucatan as well as from QRoo using the same methods. All samples were kept at -20 °C until further analyses.

### Comparison of cuticular profiles

To compare the cuticular profiles of the different species and localities we used GC–MS. For this, one microliter of each extract in hexane was placed in the inlet port of an Agilent Technologies gas chromatograph (7890) coupled with a mass spectrometer (5975C). The inlet in splitless mode was set at 300 °C (splitless time, 1.5 min). A fused-silica column HP-5MS (5% phenyl-95% polydimethylsiloxane; 30 m, 0.25 mm ID, 0.25 µm) was employed with helium (purity 99.99) carrier gas at 1.0 mL/min. The oven program started at 40 °C and reached 300 °C at 10 °C/min with a holding of 13 min. The eluent was transferred into the MS detector via a transfer line held at 300 °C with 3 min of solvent delay. Typical conditions of MS detector were optimized through the autotune software option. The electron impact mode (70 eV) was used as an ionization source (230 °C) and masses were monitored between 25 and 525 *m/z*. The temperature of the quadrupole was 150 °C. The total analysis time was 39 min.

The relative contribution of each cuticular hydrocarbon (alkanes and alkenes) was calculated based on the average ion current peak areas obtained for colonies of each species. The assignment of chromatographic peaks was accomplished by comparison of the experimental mass spectra with the spectra in the NIST data base^20^. Also, retention indices were obtained for each peak using reference samples containing n-alkanes (C10 to C30).

We focused further analyses on unsaturated cuticular hydrocarbons, which are known to serve as recognition cues in stingless bees^15^.

To test for chemical similarity between *Lestrimelitta* sp. with *L. niitkib* and potential hosts, we submitted the data of peak areas of each unsaturated hydrocarbon (all 14 alkene isomers detected) to a principal component analysis (PCA). We then used these new PCs as variables in separate analyses to establish relationships between groups^21^. PCA scores were calculated for the first three components that included the largest amount of variation in the data. We also compared the scores among species and localities using a GLM analysis (with Bonferroni correction)^22^. The scores for the first two components were also used to produce plots of the corresponding values for each colony and species onto a bidimensional scale.

### Morphometric comparisons

For the comparison of size and shape of the samples collected from different species and localities, we analyzed the morphometrics of meristic characters and the geometry of the forewing.

For comparison of meristic characters, the head, thorax, right forewing and right hind leg of ten workers from each sample were dissected and mounted on slides using routine procedures. The structures were photographed using a Leica S8 APO microscope and four characters related to bee size were measured using ImageJ^23^ on each worker: head width, intertegular distance, forewing length, and femur length^24^. A General Linear Model (GLM) in the statistical software SAS^22^ was used to compare the size of the different structures between species and localities. Additionally, a PCA was used to obtain parameters of overall body size combining the four meristic traits measured on individual bees. The resulting coefficients for the first three PCs were used to calculate scores as individual measures of body size and were compared using a GLM in SAS^22^.

The shape and size of the forewing was also compared between *Lestrimelitta* sp. and *L. niitkib* using geometric morphometrics. Twelve intersections of the forewing veins (Supplementary files, Fig. S2 online), were established as homologous landmarks using tpsDig2 software version 2.12^25^. The coordinates of the landmarks were Procrustes fitted to evaluate existing shape variation using the software MorphoJ version 1.07a^26^. Within the MorphoJ software, further statistical computations including Procrustes ANOVA, Discriminant Function Analysis (DFA), Principal Component Analysis (PCA) and multivariate regression analyses were conducted to discriminate between bee types^27^.

### DNA barcoding

Genetic differences among *Lestrimelitta* sp*.* and *L. niitkib* were analysed by comparing the DNA barcode region of the mitochondrial gene cytochrome oxidase I or *Cox*1. Barcoding is an accepted technique at discriminating species in almost all groups of animals^28^ and has been successfully applied for such purposes in stingless bees^29^. DNA was extracted from one individual per colony using a Chelex protocol and amplified with universal PCR primers for animal barcoding^30^ using standard methods as part of the CBol initiative to barcode the bees of the world^31^. Phylogenetic and molecular evolutionary analyses were conducted using MEGA version 11^32^. In short, sequences were aligned, the best model for sequence evolution tested, and then sequences were compared using neighbor joining (NJ) and maximum likelihood (ML) after correction for the best model: the Tamura 3-parameter model with rate variation among sites modelled using a discrete gamma distribution (T92 + G). Two sequences of *Lestrimelitta danuncia* from Costa Rica (BOLD database, kindly provided by L. Packer) were included as representative congeners as was one of the stingless bee *Plebeia frontalis* (downloaded from the NCBI database), as an outgroup.

### Taxonomic identification

As a final step, specimens of *Lestrimelitta* sp*.* and *L. niitkib*, were compared using traits that are discriminatory in the taxonomy of bees of this genus, namely, the shape of the propodeal spiracle, length of the mesotibial spur, presence or absence of hairs on the body, and the length, density, and type of pubescence^33,34^. To date, two large species groups can be recognized within *Lestrimelitta* based on the shape of the propodeal spiracle: the exclusively South American *L. limao* (Smith, 1863) species group, which consists of species with an ovoid propodeal spiracle, 2–3 times longer than broad^35,36^, and the *L. ehrhardti* (Friese, 1931) species group found in both Central and South America, which are distinguished by an elongate propodeal spiracle, at least 4.6 times longer than broad^35^.

## Results

### Comparison of cuticular profiles

**Figure 1:**
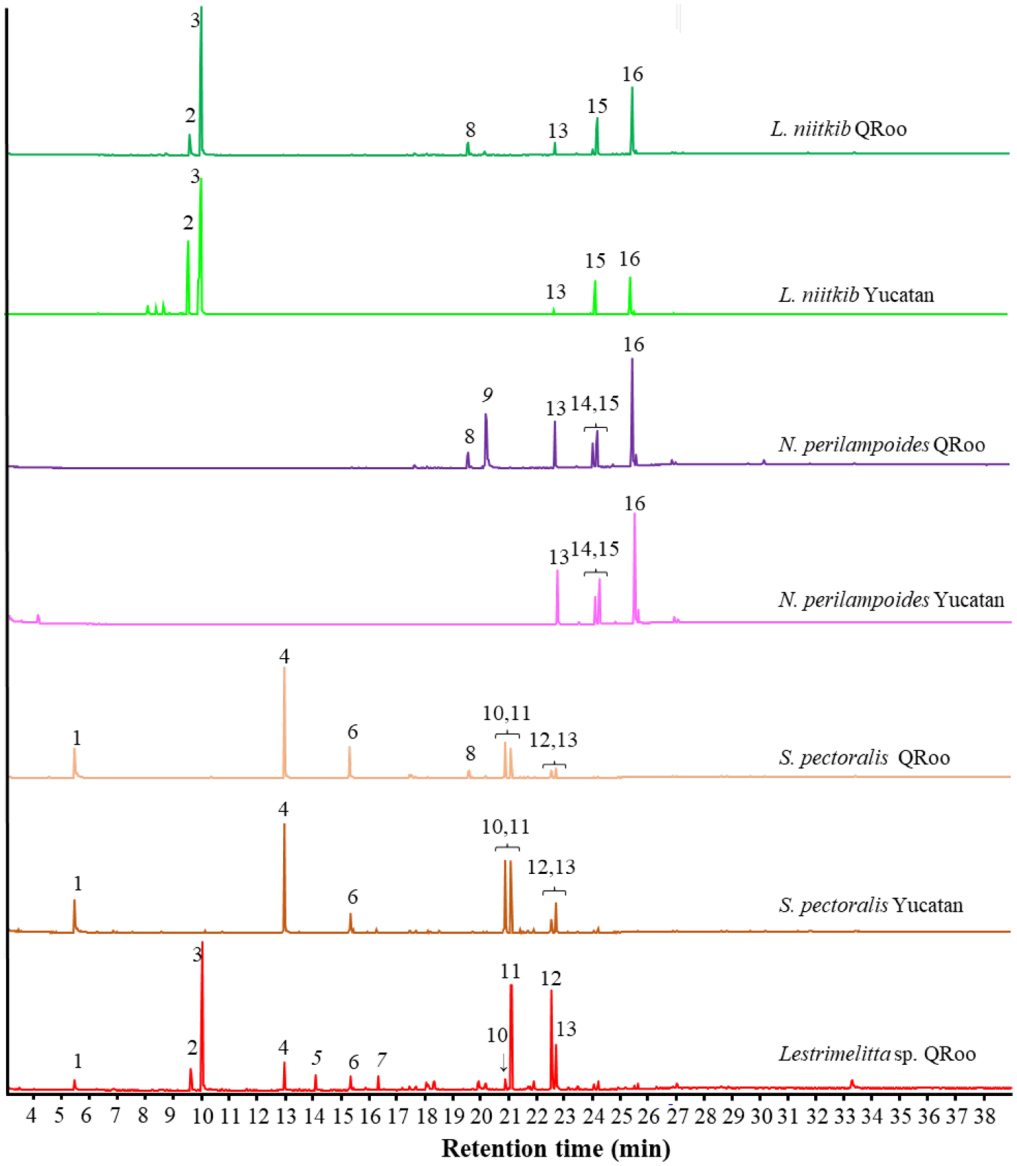
Typical GC/MS chromatograms of cuticular extracts of stingless bee species and localities from the Yucatan Peninsula. (1) benzaldehyde, (2) Z-Citral, (3) E-Citral, (4) 2-tridecanone, (5) not identified, (6) 2-pentadecanone, (7) not identified, (8) methyl octadecanoate, (9) not identified, (10) tricosene, (11) tricosane, (12) pentacosene, (13) pentacosane, (14) heptacosene, (15) heptacosane, (16) nonacosene.

**Figure 2:**
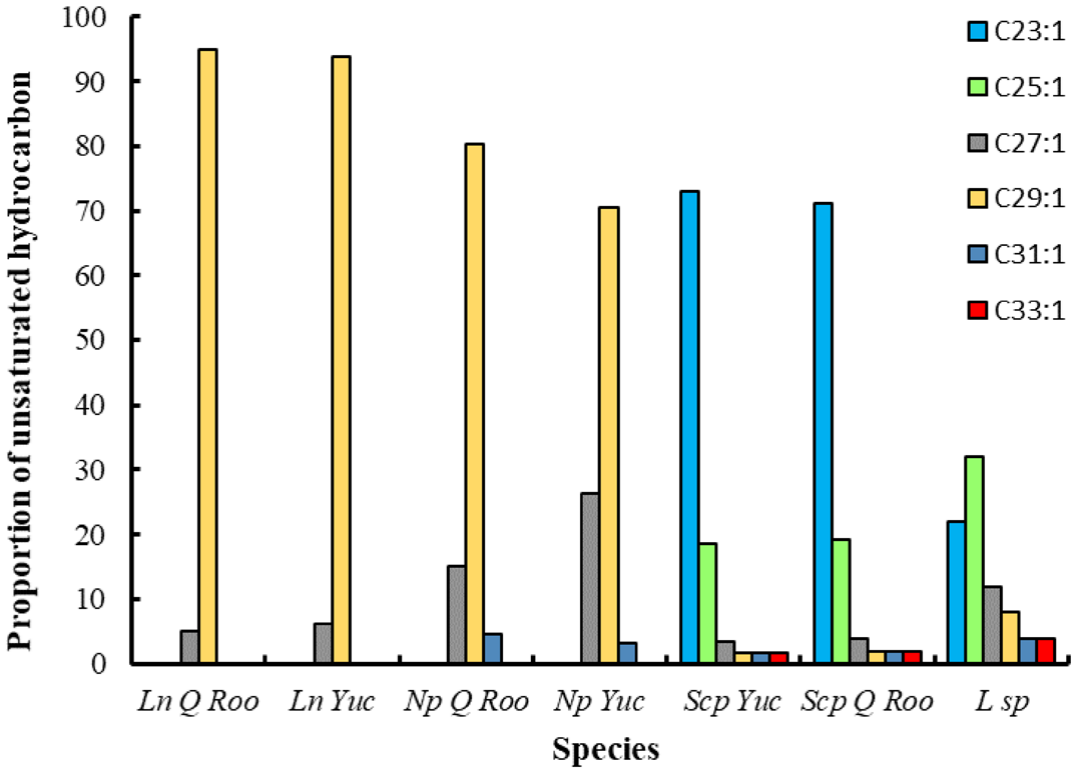
Proportion of unsaturated cuticular hydrocarbons present in *Lestrimelitta* sp*., L. niitkib* and potential host species from Yucatan and Quintana Roo. Ln, *L. niitkib*; Np, *N. perilampoides*; Scp, *S. pectoralis*; *Yuc* Yucatán; *Q Roo* Quintana Roo; L sp, *Lestrimelitta* sp.

A variety of linear alkanes and alkenes were found in the studied species in the range of carbon lengths of C19 to C33 (Figs. 1 and 2). Alkadienes and branched alkanes were not detected in any species (Fig. 1 and Supplementary files Table S1 online). If more than one alkene isomer was present, as indicated by differences in the retention time, they were numbered accordingly (Supplementary files, Table S1 online). The comparison of whole chromatograms (Fig. 1) and the graphs of relative alkene proportion (Fig. 2) showed obvious differences between *Lestrimelitta* sp*.* and *L. niitkib* from both Yucatan and Q Roo.

PCA further confirmed these differences. The first three components for the alkene based PCA encompassed 76% of the variance in the data, with PC1 alone explaining 42% of the variation (Supplementary files, Table S2 online). The compounds with highest correlation coefficients with PC1 were the three isomers of C25:1 followed by the three isomers of C23:1. For PC2, the compounds with highest correlations were the third isomer of C25:1 and the first isomer of C29:1 (Supplementary files, Table S3 online).

**Figure 3:**
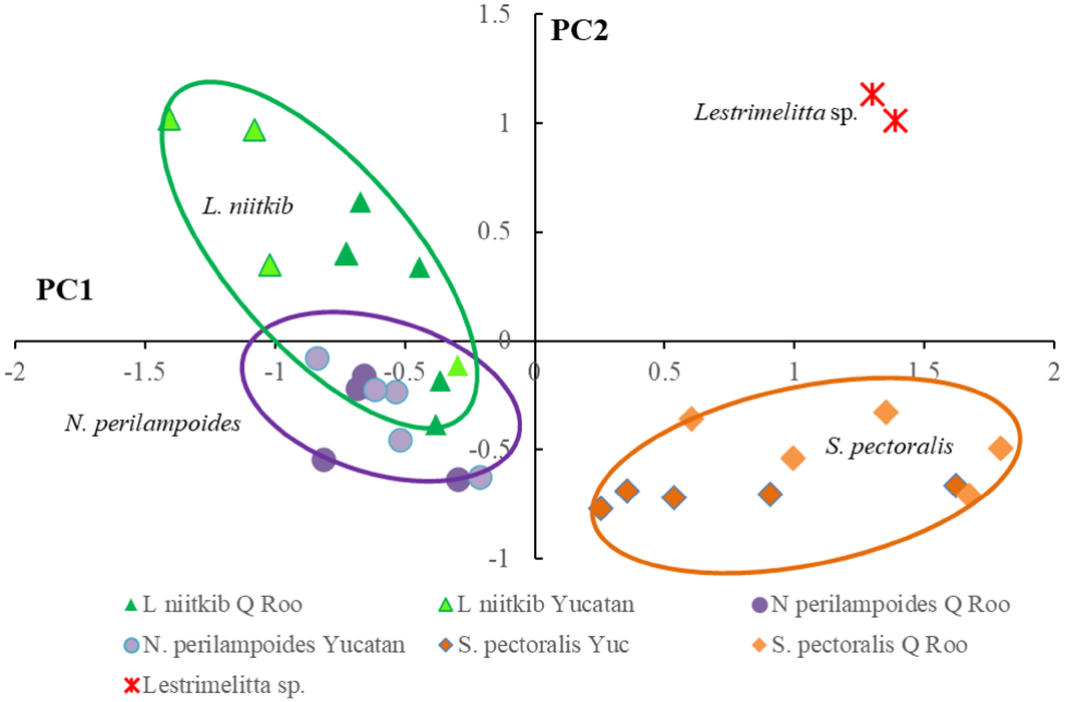
Distribution of alkene derived Principal Component (PC) scores of *Lestrimelitta* sp*.* and different species and populations of stingless bees from the Yucatan Peninsula against PC1 and PC2.

Colony scores were obtained by means of the corresponding coefficients for each PC and further compared by means of a GLM analysis (Supplementary files, Table S4 online). The results of both the GLM analysis and the plot of scores against PC1 and PC2 (Fig. 3) revealed significant statistical differences between *Lestrimelitta* sp*.* and *L. niitkib* from Yucatan and QRoo and also with *N. perilampoides* from both states. On the other hand, scores for PC1 of *S. pectoralis* from both Yucatan and QRoo were not different to *Lestrimelitta* sp. (Fig. 3, Supplementary files Table S4 online), confirming that their similarity was mainly due to C25:1 and C23:1. *Lestrimelitta* sp. and *S. pectoralis* did differ in PC2 scores, suggesting differentiation in other components of their alkene bouquet, namely the third isomer of C25:1 and the first isomer of C29:1 (Fig. 3) Chemical distances were also compared among the bee species and populations, which provided further support for the differences found in the PCA (Supplementary files, Fig. S3 online).

### Morphometric comparisons

**Figure 4:**
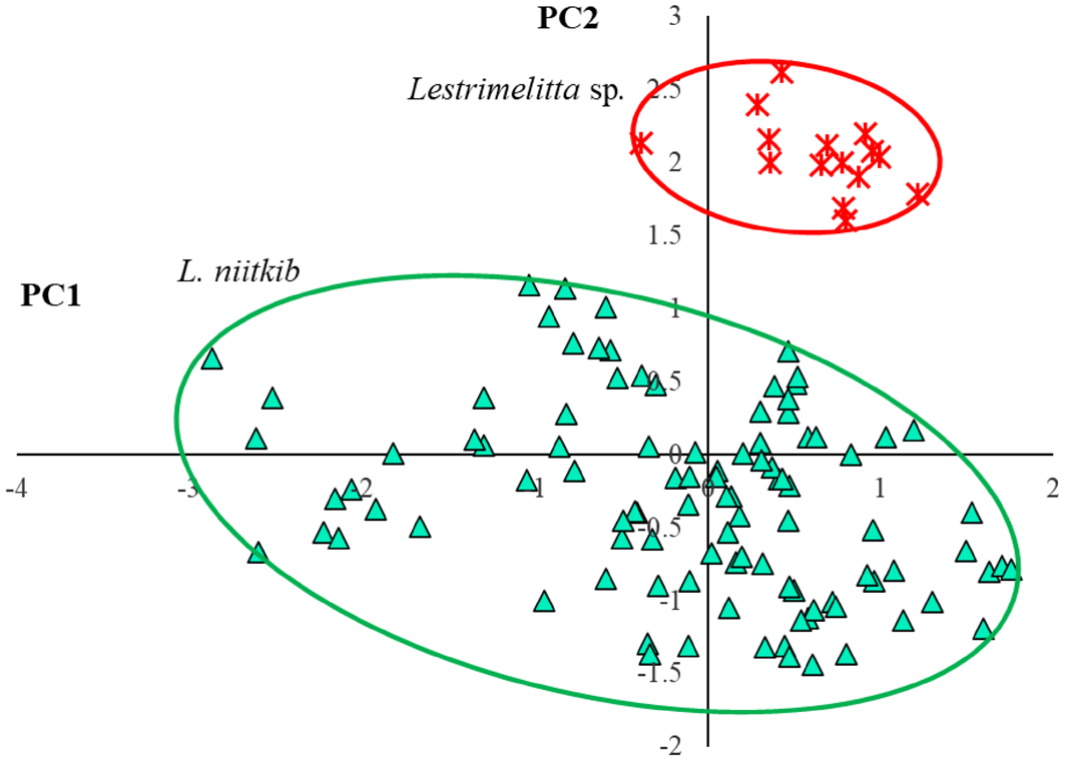
Distribution of *Lestrimelitta* sp. (asterisks) and *L. niitkib* (triangles) scores derived from a principal component (PC) analysis of four meristic characters plotted against PC1 and PC2.

Given the chemical similarity between populations of *L. niitkib* from Yucatan and QRoo, individuals of this species from both localities were pooled in one group for morphometric comparison with *Lestrimelitta* sp. Comparison of the four individual meristic characters revealed two that showed significant differences in the length of the femur and forewing (Supplementary files, Table S5 online). For this PCA, the first three Components encompassed 95% of the variation in the data, with PC1 explaining 59% of the total variance (Supplementary files, Table S6 online). Head width had the highest correlation with PC1, while forewing length had the highest correlation with PC2 (Supplementary files, Table S7 online). Highly significant differences resulted when the scores of both *Lestrimelitta* species were compared for all three Components (Supplementary files, Table S5 online). The plot of individual scores against PC1 and PC2 confirmed the morphometric separation of *Lestrimelitta* sp*.* and *L. niitkib* along PC2, indicating that differences were due to the length of the forewing (Fig. 4).

**Figure 5:**
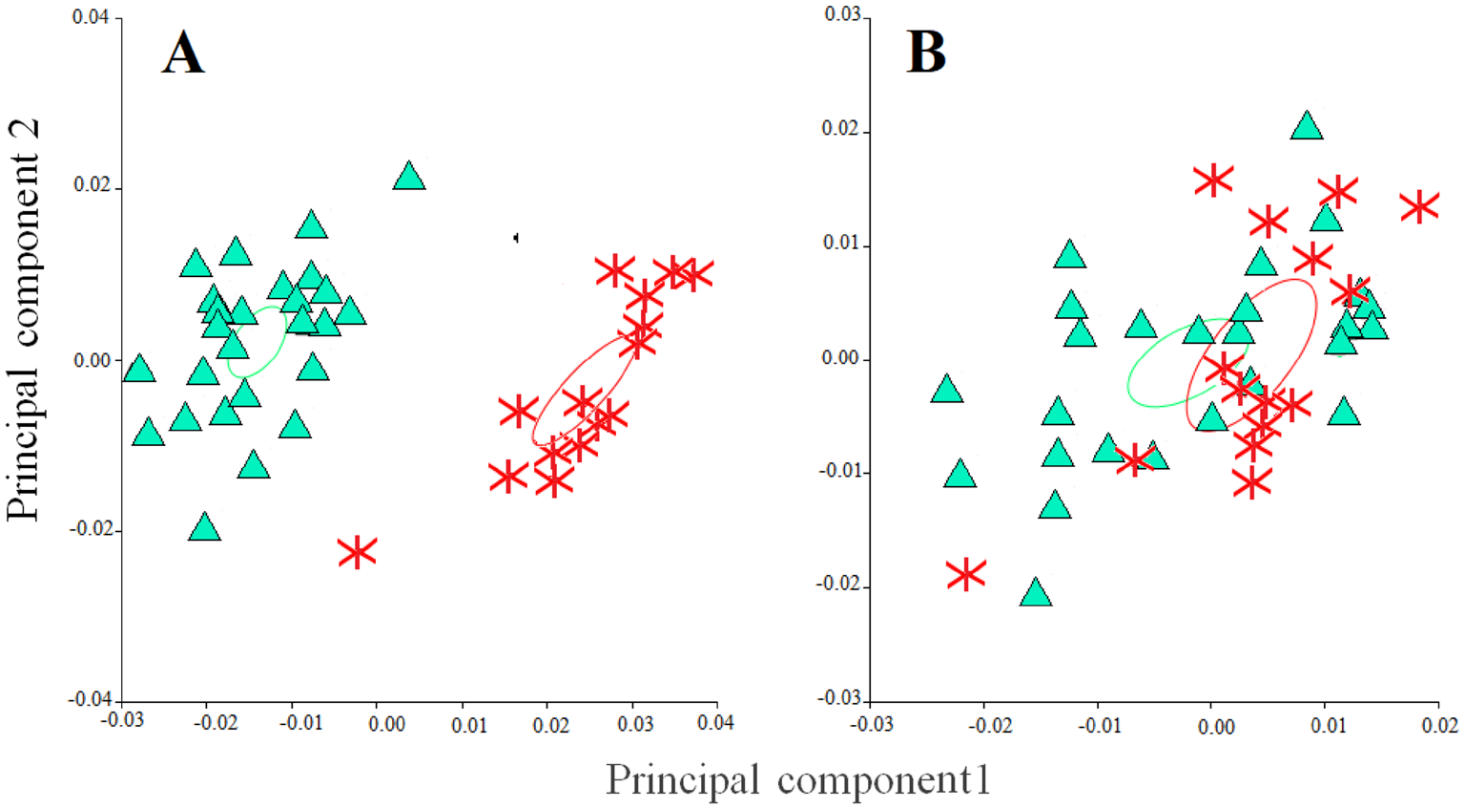
Plot of the Procrustes values for *L. niitkib* (triangles) y *Lestrimelitta* sp. (asterisks) against components 1 and 2, including 90% confidence ellipses, in: **(A)** with the effect of size and **(B)** regression corrected to represent differences in the shape of the forewing.

Geometric morphometrics of the forewings confirmed these PCA-based results. The Procrustes ANOVA (Supplementary Files, Table S3) revealed significant differences in size; the forewings of *Lestrimelitta sp*. were significantly larger (centroid size: X̄ ± DS = 594.59 ± 8.09) compared with those of *L. niitkib* (centroid size: X̄ ± DS = 523.51 ± 16.03). The DFA also showed significant differences in the shape of the forewing (Procrustes distance = 0.034, T^2^ = 1086.87, P ≤ 0.001, permutation at 1000 iterations); mean shapes of forewings of both species are each displayed by a wireframe (Supplementary Fig. S3 online). Notably, the PCA showed that the majority of the shape variation in the forewings was explained in the first three dimensions, accounting for 71% of the total variance (variance explained: PC1 = 53%; PC2 = 12%; PC3 = 6%) (Fig. 5A). After allometric correction through the covariance matrix of the residuals of the regression, the shape variation decreased considerably, accounting for 51% of the total variance (variance explained: PC1 = 25%; PC2 = 17%; PC3 = 9%) (Fig. 5B). This multivariate regression showed high allometric influence of 39.18% (p ≤ 0.0001), meaning that most of the difference in shape between the forewings of both species is due to their size.

### DNA barcoding

**Figure 6:**
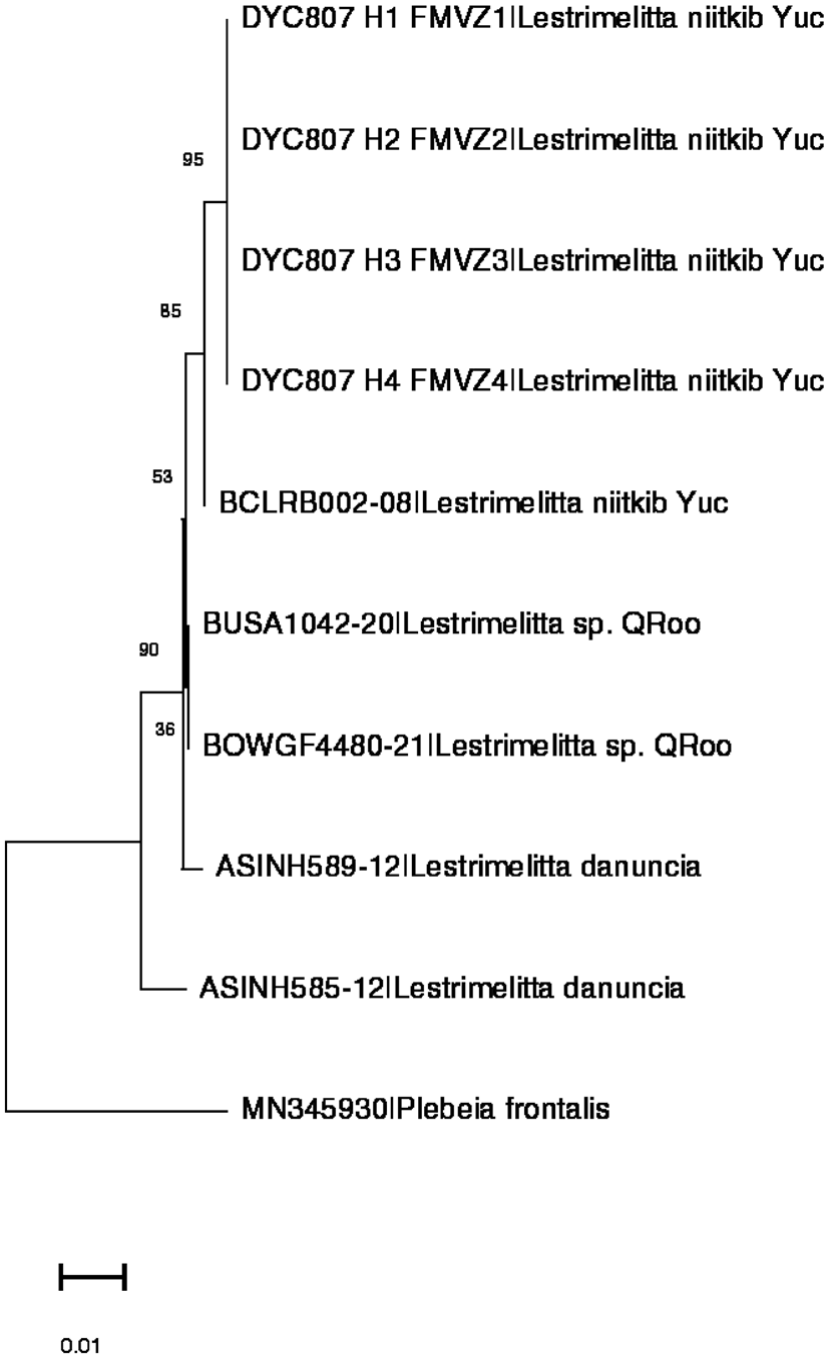
NJ tree obtained from the analysis of the fragment *Cox* 1 of the mt DNA of *Lestrimelitta* specimens from the Yucatán Peninsula (*Yuc*  Yucatan State; *QRoo* Quintana Roo State) and from Costa Rica (*Lestrimelitta danuncia*), with *Plebeia frontalis* as outgroup. Values show bootstrap branch support (500 replicates). The bar represents nucleotide sequence divergence.

The sequencing of the *Cox 1* region of mtDNA generated a final dataset of 603 bases, and allowed assignment of the samples of *Lestrimelitta* from the Yucatán Peninsula according to their genetic similarity to species already in the BOLD database^37^. The resulting NJ tree assigned the specimen of *L. niitkib* as expected, together (Fig. 6). However, the specimen of *Lestrimelitta* sp*.* was not assigned to the latter branch, but to *L. danuncia*, a species not yet reported in Mexico^38^, though we note weak bootstrap support for the branch (see also ML tree in supplementary file Fig. S4 online).

The two *Lestrimelitta* sp. from QRoo were identical, 0 bp difference. The two *L. danuncia* samples had 10 pb differences (variability within the species = 10/603 or 0.017). The two *Lestrimelitta* sp. from QRoo differed from the two *L. danuncia* samples by on average 5 bp (divergence to danuncia = 5/603 or 0.008). In detail, the two *Lestrimelitta* sp. QRoo differed from the *L. danuncia* samples by 2 bp and 8 bp (i.e. (2 + 8)/2 = average 5 bp).

### Taxonomic identification

**Figure 7:**
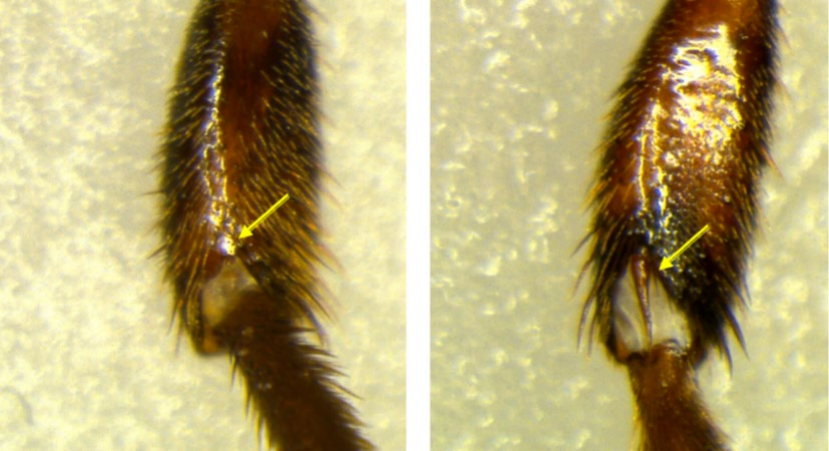
Detail of the tibial spur (pointed with a yellow arrow): minute in *L. niitkib* (left) and elongated in *Lestrimelitta* sp. (right), similar to that of *L. danuncia*.

The species performing the attacks on *S. pectoralis* in QRoo belongs to the *Lestrimelitta* species group with an elongated spiracle of the propodeum, the *ehrhardti* group of species^33^ (Supplementary files, Fig. S5 online). Currently 13 species are recognized within this group. Noteworthy, the two *Lestrimelitta* reported from Mexico belong to this group: *L. chamelensis* and *L*. *niitkib*^38^. However, both species have a greatly reduced mesotibial spur, in strong contrast with *Lestrimelitta* sp*.* which has an elongated one (Fig. 7). This bee has a long mesotibial spur, similar to *L. danuncia* (currently not reported from Mexico). In addition, there are subtle differences in the setae on the anterior edge of the mesoscutum between *Lestrimelitta* sp. and *L. danuncia* (data not shown). Such differences could represent regional variation or a new species, sister to *L. danuncia*, as also the barcoding suggests. Presently, *Lestrimelitta* sp. has only been found in QRoo, but it represents a new report for Mexico.

## Discussion

In this study, the unusual attacks of *Lestrimelitta* to a seemingly non-preferred host provided the opportunity to test the relative importance of chemical deception in these obligate cleptobionts. GCMS analyses, complemented with genetic and taxonomic tools, led to the identification of an unreported species for México. Importantly, this is the first record of a chemical comparison between sympatric cleptobionts or cleptoparasites species within the same genus. The results, a) reveal that different species of *Lestrimelitta* resemble the alkene profiles of their preferred hosts, which implies that chemical deception seems to be common in these organisms, b) that the cleptobiont chemical profiles are species-specific and, c) that, in sympatric species, chemical profiles may relate to the differential selection of hosts, thus possibly serving to avoid resource competition and support niche separation. These results have several implications.

Our study extends the findings of chemical cleptobiont-host similarity found in *L. niitkib* to a possible new species of *Lestrimelitta*, adding support to the notion of chemical deception as a mechanism used by obligate stingless bee cleptobionts to select and invade their hosts. Chemical similarity between *Lestrimelitta* and its preferred hosts could help scouts to avoid nest guards without eliciting an aggressive response, a common strategy also used by other social parasites^39,40^. In contrast, species with chemically distant profiles react rapidly to *Lestrimelitta* intruders, which invariably leads to their elimination^11^, thus preventing attacks to their colonies. However, in chemically similar species, *Lestrimelitta* scouts may enter the host colony and collect information for the recruitment of nestmates.

Noteworthy, *Lestrimelitta* does not show an exact cuticular profile (chemical crypsis) with that of their host, as occurs in cleptoparasites that live inside their host’s nest^41–43^. Thus, this may be better classified as a case of chemical masquerade^43^. The relationship of *Lestrimelitta* with its hosts is temporary, and chemical deception probably becomes only useful to avoid initial detection during the first stages of their raids. Under this scenario, evolving towards exact mimicry of a single host (crypsis) would probably be too costly to the already specialized narrow niche of obligate cleptobiosis in a highly eusocial organism^1^. Instead, it would be more advantageous to own a profile resembling several hosts, which could help to better deal in case of changes in host diversity and abundance^11^.

One key aspect in the dynamics of this system is understanding how *Lestrimelitta* comes to chemically resemble their hosts. It has been suggested that chemical similarities found between *L. niitkib* and its preferred hosts, *N. perilampoides* and *Plebeia,* may derive from phylogenetic relatedness^11^. Alternatively, like other cleptoparasites, *Lestrimelitta* could produce cues that match the host's chemical profile^44^, or acquire them by exposure to the nest or host environment^45^.

Presently, it is difficult to propose a clear origin of the cuticular compounds used in host deception*,* but since *Lestrimelitta* uses the larval food and nest materials of their hosts, it is likely that both could serve as sources of chemical cues. Larval food shows contrasting variation among and within species of stingless bees, presumably derived from the different floral sources exploited^46^. However, when pollen of different sources was fed to adult *Frieseomelitta* stingless bees, no change of their cuticular profile occurred^47^. Likewise, larval food from different colonies used to rear gynes of *S. pectoralis* did not produce significant differences in the cuticular profiles of the emerging adults^48^. Although these experiments suggest that food may not be the origin of intraspecific CHC chemical differences in stingless bees, its effect in interspecific cross-fostering experiments (reproducing the model of *Lestrimelitta*-hosts) has not been analyzed.

The cerumen (a mix of beewax and plant resins) robbed by *Lestrimelitta* from host colonies to build their nest structures could also be a source of cuticular odours. Cerumen has been acknowledged as an important source of chemicals used in recognition by *Frieseomelitta* stingless bees. Individuals confined in contact with the cerumen of a foreign nest were quickly rejected when placed back in their own nest^47^. However, in *Tetragonisca angustula*, no significant rates of rejection occurred when individuals were put in contact with cerumen from their own or a foreign nest^49^. It is known that stingless bee species incorporate resin compounds in their own cuticular profile, thereby enriching their chemical diversity^50^. However, the bioassays performed so far are not conclusive and more work is needed so as to assess the role of cerumen and resin in stingless bee recognition.

Another way by which *Lestrimelitta* may acquire the host’s odours is by physical contact. Such a mechanism has been assumed in myrmecophilous insects, which, similarly to *Lestrimelitta,* only partially mimic their hosts^40,51^. In the course of nest attacks, *Lestrimelitta* comes into contact with hosts and host nest materials, making it possible to acquire odours of the raided species in the process. However, although the exchange of surface compounds through physical contact has been previously demonstrated among social insect nestmates^52^, evidence for its occurrence among parasites and hosts is limited^53^.

One important finding was that the cuticular profile of the recently identified *Lestrimelitta* sp. is qualitatively and quantitatively different to that of sympatric *L. niitkib* (in particular in the proportions of alkanes and alkenes). Distinct, species-specific chemotypes could prove useful in the chemotaxonomy of cleptobiotic bees^54^. Notably, the profiles of the two *Lestrimelitta* in this study closely resembled their specific hosts. The profile of *L. niitkib* resembled that of *N. perilampoides*, its preferred host^11^, while that of *Lestrimelitta* sp*.*, resembled the profile of *S. pectoralis*, but it was different to *N. perilampoides*. This apparent selection of host species in relation to differences in the chemical profile of the cleptobionts is remarkable because, from a common strategy to overcome their host chemical detection systems, modifications could have evolved so that different species of *Lestrimelitta* chemically resemble their respective hosts. Thus, each sympatric species of cleptobiont might be chemically coevolving with a group of hosts. If so, cuticular cues involved in host selection could be key traits in the differentiation of *Lestrimelitta* species or chemotypes. In insects, adaptation to novel environments can involve changes in cuticular hydrocarbons that could lead to environmentally based divergent selection^16,55^. Mechanisms of sympatric speciation through intraspecific social parasitism have been proposed for the evolution of Hymenopteran workerless parasites^56^ and cryptic species divergence in ants^57^. Our model system could be driven by competition for resources among cleptobionts, i.e. host shifts. A parasite-host race emergence, as in other cases of sympatric speciation^57–59^, could have profound effects in the evolution of stingless bees.

In conclusion, by chemically exploiting species-specific hosts, sympatric cleptobionts may be under a type of ecological adaptation. If such adaptation has a genetic basis, it may eventually lead to their reproductive isolation^60^. In stingless bees, gene flow between populations could be further reduced because of their philopatric mode of reproduction and short colony dispersal, reinforcing genetic differentiation^61^.

A note should be made on the high abundance of citral in both species of *Lestimelitta* in this study. Propaganda substances produced in the mandibular and labial glands of the cleptobiont are known to have different effects on hosts, from disruption to retreat, and thus seems to play a key role in the process of host raiding^2,62^. Similarly, some host species seem to be raided by the use of sheer force^2^. Therefore, it is likely that adaptation to evade detection by chemical means could be one of various strategies which *Lestrimelitta* may use to exploit different species of stingless bee or at different stages during raids^11^.

Our study leaves many open questions which we hope will encourage investigation of the scarcely studied relationship between stingless bees and cleptobionts. Regions with a diversity of sympatric *Lestrimelitta* species may be ideal to test the chemical deception hypothesis. Empiric evidence is required on how chemical convergence between cleptobiont and host arises, as well as the role of different compounds, propaganda substances and their mixtures in deception. It is key to determine how chemical resemblance is acquired, and thus if there is an arms race between host and cleptobionts, which may differ under sympatry *versus* allopatry. Answers to these questions will help to deepen our knowledge of mutualistic interactions and species divergence, and to understand the importance of cleptobionts in the evolution and health of rich and varied stingless bee communities. This could improve their image as pests of stingless beekeeping and stop the destruction of their colonies^61^.

## Supplementary Information

Supplementary Information.

## Data Availability

JJGQE is the corresponding author from whom materials can be requested.
