# Supplementary material for: Sympatric cleptobiotic stingless bees have species-specific cuticular profiles that resemble their hosts
Source: Sci Rep. 2022 Feb 16;12:2621. doi: 10.1038/s41598-022-06683-w (PMC8850540; doi:10.1038/s41598-022-06683-w)
Supplement: Supplementary file 1 — Supplementary Information. [file 41598_2022_6683_MOESM1_ESM.docx]

**
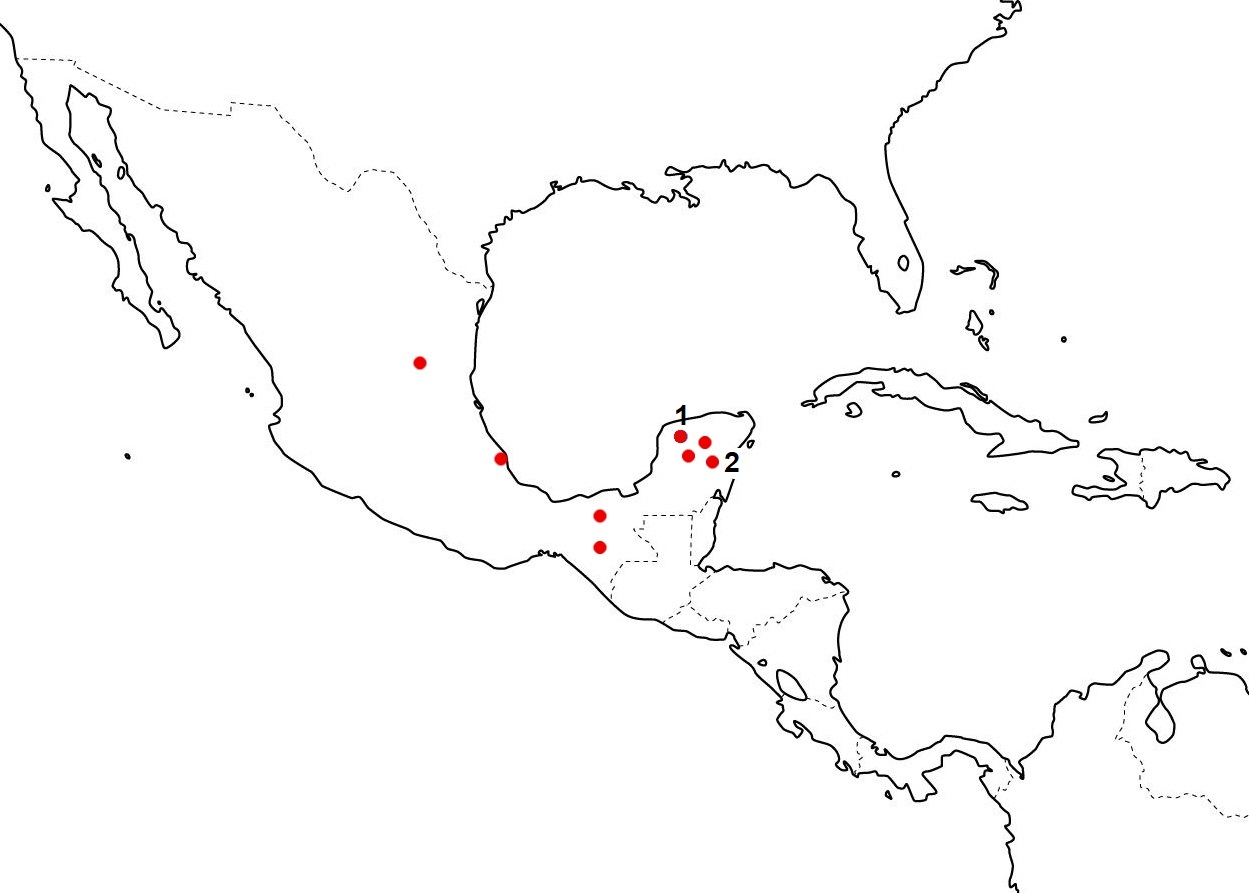
**

**Fig. S1. Map on Mexico presenting in red circles the localities where *Lestrimelitta niitkib* has been reported [from 36,61]. Numbers 1 and 2 on the Yucatan Peninsula, represent the localities of Merida and Felipe Carrillo Puerto, respectively, where samples for this study were collected. The locality of Felipe Carrillo Puerto in Quintana Roo is the where the not yet reported in Mexico *Lestrimelitta* spp. has been found.**

**
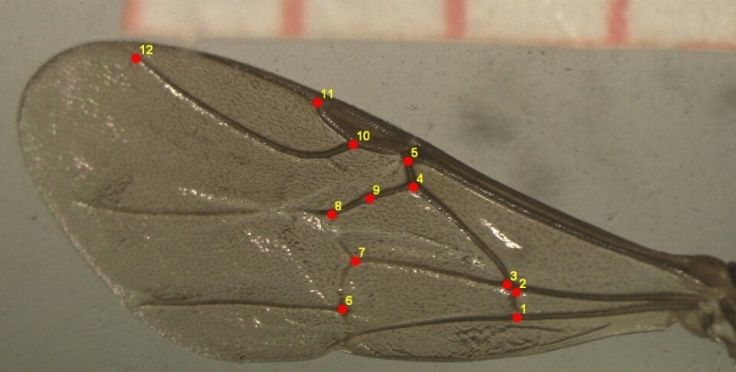
**

**Fig. S2. Pattern of *landmarks* on the forewings of *Lestrimelitta* used for the study of geometric morphometrics.**

**Table S1. Relative proportions of cuticular hydrocarbons found in *L.* *niitkib*, *Lestrimelitta* *sp.* and potential hosts of Quintana Roo and Yucatán.**

|  | | | **Quintana Roo** | | | **Yucatán** | | |  |
| --- | --- | --- | --- | --- | --- | --- | --- | --- | --- |
| **Analyte** | **RT** | **RI** | ***Ln*** | ***Np*** | ***Scp*** | ***Ln*** | ***Np*** | ***Scp*** | ***Lestrimelitta sp.*** |
| **Nonadecane**  **(C19)** | 17.695 | 1862 | - | 0.0125 (0.004) | 0.020 (0.003) | - | 0.001  (0.002) | 0.004 (0.003) | - |
| **Heneicosane**  **(C21)** | 19.717 | 2109 | - | 0.005  (0.001) | 0.01  (0.005) | - | 0.001  (0.001) | 0.004 (0.001) | - |
| **Tricoseno-1**  **(C23:1)** | 20.894 | 2235 | - | - | 0.398 (0.04)2 | - | - | (0.346)  (0.06) | 0.12 |
| **Tricoseno-2**  **(C23:1)** | 20.95 | 2239 | - | - | 0.033 (0.004) | - | - | 0.030 (0.007) | 0.005 |
| **Tricoseno-3**  **(C23:1)** | 21.034 | 2244 | - | - | 0.008 (0.0001) | - | - | 0.002 (0.0005) | 0.007 |
| **Tricosano**  **(C23)** | 21.098 | 20.48 | 0.003  (0.002) | 0.004 (0.001) | 0.274 (0.039) | 0.001 (0.0001) | 0.004 (0.001) | 0.290 (0.035) | 0.207 |
| **Pentacoseno-1**  **(C25:1)** | 22.545 | 2436 | - | - | 0.091 (0.021) | - | - | 0.074 (0.019) | 0.303 |
| **Pentacoseno-2**  **(C25:1)** | 22.59 | 2439 | - | - | 0.023 (0.004) | - | - | 0.024 (0.005) | 0.05 |
| **Pentacoseno-3**  **(C25:1)** | 22.657 | 2444 | - | - | - | - | - | - | 0.019 |
| **Pentacosano (C25)** | 22.715 | 2448 | 0.053 (0.024) | 0.117 (0.042) | 0.090 (0.017) | 0.042 (0.003) | 0.172 (0.019) | 0.126 (0.021) | 0.135 |
| **Heptacoseno-1**  **(C27:1)** | 23.163 | nc | - | - | - | - | - | - | 0.008 |
| **Heptacoseno-2**  **(C27:1)** | 23.483 | nc | 0.01 (0.001) | 0.006 (0.001) | 0.003 (0.0001) | 0.01 (0.001) | 0.005 (0.001) | 0.003 (0.0001) | 0.008 |
| **Heptacoseno-3**  **(C27:1)** | 24.059 | 2636 | 0.020 (0.001) | 0.081 (0.055) | 0.011 (0.004) | 0.014 (0.003) | 0.14 (0.037) | 0.013 (0.005) | 0.016 |
| **Heptacoseno-4**  **(C27:1)** | 24.105 | nc | 0.006 (0.001) | 0.014 (0.006) | 0.003 (0.001) | 0.005 (0.001) | 0.10 (0.006) | 0.005 (0.001) | - |
| **Heptacosano**  **(C27)** | 24.233 | 2647 | 0.314 (0.02) | 0.130 (0.01) | 0.013 (0.001) | 0.413 (0.03) | 0.144 (0.02) | 0.028 (0.001) | 0.023 |
| **Nonacoseno-1**  **(C29:1)** | 24.931 | nc | 0.004 (0.001) | 0.001 (0.001) | 0.004 (0.005) | 0.003 (0.001) | 0.001 (0.001) | - | 0.008 |
| **Nonacoseno-2**  **(C29:1)** | 25.2 | nc | 0.001 (0.001) | - | 0.019 (0.001) | - | 0.001 (0.0001) | - | 0.003 |
| **Nonacoseno-3**  **(C29:1)** | 25.481 | 2836 | 0.545 (0.028) | 0.517 (0.071) | 0.006 (0.001) | 0.446 (0.038) | 0.432 (0.028) | 0.007 (0.001) | 0.008 |
| **Nonacoseno-4**  **(C29:1)** | 25.5 | nc | 0.003 (0.001) | 0.013 (0.013) | - | 0.016 (0.0001) | - | 0.001 (0.0003) | - |
| **Nonacosano (C29)** | 25.619 | 2846 | 0.021 (0.004) | 0.045 (0.013) | 0.008 (0.003) | 0.032 (0.006) | 0.040 (0.006) | 0.008 (0.002) | 0.016 |
| **Hentriaconteno**  **(C31:1)** | 26.882 | nc | 0.006 (0.002) | 0.031 (0.01) | 0.008 (0.003) | 0.004 (0.0001) | 0.021 (0.008) | 0.011 (0.004) | 0.008 |
| **Hentriacontano**  **(C31)** | 27.016 | nc | 0.007 (0.007) | 0.016 (0.008) | 0.003 (0.003) | 0.009 (0.002) | 0.011 (0.002) | 0.006 (0.003) | 0.019 |
| **Tritriaconteno**  **(C33:1)** | 28.647 | nc | - | - | 0.009 (0.003) | - | - | - | 0.01 |

**RT**: Retention time; **RI**: Retention index; **Ln**: *Lestrimelitta* *niitkib*; **Scp**: *Scaptotrigona* *pectoralis*; **Np**: *Nannotrigona* *perilampoides*; **nc**: no concentration detected

**Table S2. Eigenvalues, proportion of variance explained by each Component of PCA for alkenes and their cumulative proportion.**

Eigenvalue Difference Proportion Cumulative

1 6.65309213 3.51468396 0.4158 0.4158

2 3.13840817 0.77631252 0.1962 0.6120

3 2.36209565 0.45792875 0.1476 0.7596

4 1.90416690 1.13916203 0.1190 0.8786

5 0.76500487 0.31322856 0.0478 0.9264

6 0.45177631 0.21768697 0.0282 0.9547

7 0.23408933 0.07053130 0.0146 0.9693

8 0.16355803 0.04475230 0.0102 0.9795

9 0.11880574 0.03065315 0.0074 0.9869

10 0.08815259 0.02441380 0.0055 0.9924

11 0.06373879 0.03464185 0.0040 0.9964

12 0.02909694 0.00637841 0.0018 0.9982

13 0.02271854 0.01764636 0.0014 0.9997

14 0.00507218 0.00484834 0.0003 1.0000

15 0.00022384 0.00022384 0.0000 1.0000

16 0.00000000 0.0000 1.0000

**Table S3. Standardized scoring coefficients for each alkene for the first four Principal Components (PC) of the PCA. The different isomers (those in supplementary material table 1) are represented with different letters next to them.**

Compound PC1 PC2 PC3 PC4

C23:1a 0.11911 -0.10766 0.01678 0.25115

C23:1b 0.12003 -0.09827 0.02388 0.25300

C23:1c 0.13318 0.11861 0.07318 0.03843

C25:1a 0.13441 0.11737 0.01526 -0.03103

C25:1b 0.14286 0.04494 0.03965 0.09774

C25:1c 0.13569 0.48303 0.00513 -0.45217

C27:1a 0.00000 0.00000 0.00000 0.00000

C27:1b -0.07689 0.19551 0.01339 0.27713

C27:1c -0.05825 -0.03450 0.32310 -0.17356

C27:1d -0.06209 -0.00714 0.34714 0.08208

C29:1a -0.04514 0.26042 0.00238 0.14002

C29:1b 0.05748 0.05827 0.22612 -0.07330

C29:1c -0.12680 0.12469 0.06819 0.13810

C29:1d -0.08392 0.14888 -0.05259 0.29080

C31:1a -0.02000 -0.04606 0.33467 0.00620

C33:1a 0.11947 0.01862 0.14033 0.20383

**Table S4. GLM analysis of Principal Component (PC) scores one to three derived from alkene compounds**

|  | **PC1** | | **PC2** | | **PC3** | |
| --- | --- | --- | --- | --- | --- | --- |
| ***F* value** | **33.21**** | | **32.9**** | | **0.093 NS** | |
|  | **mean** | **St. err.** | **mean** | **St. err.** | **mean** | **St. err.** |
| ***L. niitkib* Q. Roo (n=3)** | **-0.530b** | **0.174** | **0.391b** | **0.155** | **-0.392** | **0.408** |
| ***L. niitkib* Yucatán (n=3)** | **-0.832b** | **0.195** | **0.582b** | **0.174** | **-0.290** | **0.456** |
| ***N. perilampoides***  **Q. Roo (n=3)** | **-0.616b** | **0.174** | **-0.353c** | **0.155** | **0.441** | **0.408** |
| ***N. perilampoides***  **Yucatán (n=3)** | **-0.544b** | **0.174** | **-0.321c** | **0.155** | **0.459** | **0.408** |
| ***S. pectoralis***  **Q. roo (n=3)** | **1.287a** | **0.123** | **-0.707c** | **0.162** | **0.321** | **0.208** |
| ***S. pectoralis***  **Yucatán (n=3)** | **0.731a** | **0.144** | **-0.485c** | **0.163** | **0.417** | **0.222** |
| ***Lestrimelitta***  ***sp* (n=2)** | **1.389a** | **-** | **1.033a** | **-** | **0.032** | **-** |

**Fig. S3. Calculation of chemical distance (cd) between *Lestrimelitta* sp. and populations of *L. niitkib* (Ln), *N. perilampoides* (Np) and *S. pectoralis* (Scp) from Yucatan (Yuc) and Quintana Roo (Q Roo).**

**
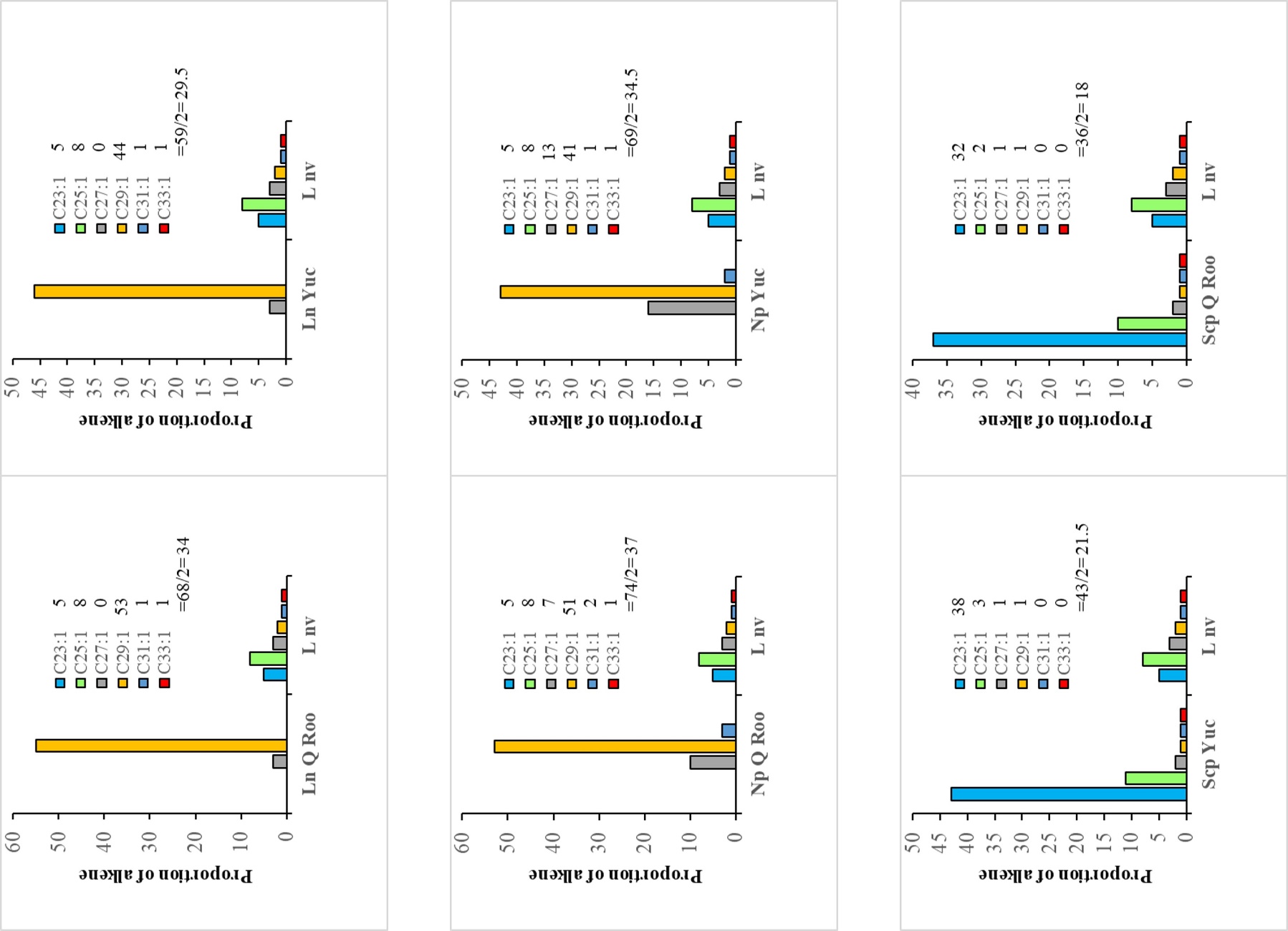
**

**Table S5. ANOVA of meristic traits measured in *Lestrimelitta sp*. and *L. niitkib* and of the scores resulting from a Principal Component Analysis for Components (PC) one to three.**

|  | **Species** | | | | |
| --- | --- | --- | --- | --- | --- |
| **Character** | ***L. niitkib* (n=100)** | | ***Lestrimelitta sp.* (n=15)** | | **F value** |
|  | **Mean** | **St err** | **Mean** | **St err** |  |
| **Head width (mm)** | 2.129 | 0.007 | 2.132 | 0.02 | 2.25 NS |
| **Intertegular distance (mm)** | 1.596 | 0.006 | 1.588 | 0.017 | 0.87 NS |
| **Forewing length (mm)** | 4.067 | 0.021 | 5.517 | 0.054 | 205.3** |
| **Femur length (mm)** | 1.259 | 0.004 | 1.281 | 0.011 | 3.34* |
| **PC1** | -0.096 | 0.097 | 0.645 | 0.251 | 7.61** |
| **PC2** | -0.306 | 0.060 | 2.046 | 0.156 | 195.4** |
| **PC3** | -0.186 | 0.087 | 1.241 | 0.227 | 34.38** |

**Table S6. Eigenvalues, proportion of variance explained by each Component of PCA for four meristic characters and their cumulative proportion.**

Component Eigenvalue Difference Proportion Cumulative

PC1 2.35952151 1.39936650 0.5899 0.5899

PC2 0.96015501 0.46185551 0.2400 0.8299

PC3 0.49829949 0.31627551 0.1246 0.9545

PC4 0.18202399 0.0455 1.0000

**Table S7. Standardized scoring coefficients for each meristic trait for the first three Principal Components (PC) of the PCA. Abbreviations are the same as used in the main tables of the text.**

Trait PC1 PC2 PC3

ID 0.32723998 -0.51921 0.64896605

HW 0.38695618 -0.2532696 -0.0682678

FWL 0.22297897 0.79954607 0.73398972

FL 0.34245296 0.26172515 -1.0209147

**Table S8. Procrustes ANOVA for centroid size and shape of forewings of *Lestrimelitta* species.**

| **Shape** | | | | | | | |
| --- | --- | --- | --- | --- | --- | --- | --- |
| **Effect** | **SS** | **MS** | ***df*** | **F** | **P(param.)** | **Pillai tr.** | **p** |
| Species | 0.0532505 | 0.00088751 | 60 | 27.58 | <.0001 | 2.34 | <.0001 |
| Individual | 0.02381027 | 0.000032 | 740 | 1.24 | 0.0012 | 10.62 | 0.0025 |
| Side | 0.00505874 | 0.00025294 | 20 | 9.78 | <.0001 | 0.9 | <.0001 |
| Ind*Side | 0.02068627 | 0.000026 | 800 | 2.85 | <.0001 | 9.74 | <.0001 |
| Error 1 | 0.01486135 | 0.0000091 | 1640 |  |  |  |  |
| **Centroid size** | | | | | |  |  |
| **Effect** | **SS** | **MS** | ***df*** | **F** | **p(param.)** |  |  |
| Species | 200404.096 | 66801.3652 | 3 | 148.75 | <.0001 |  |  |
| Individual | 16616.3284 | 449.089958 | 37 | 4.44 | <.0001 |  |  |
| Side | 128.396851 | 128.396851 | 1 | 1.27 | 0.2665 |  |  |
| Ind*Side | 4044.15267 | 101.103817 | 40 | 13.96 | <.0001 |  |  |
| Error 1 | 594.019722 | 7.244143 | 82 |  |  |  |  |

**Fig. S4. Maximum liklihood tree obtained from the analysis of the fragment *Cox* 1 of the mt DNA of *Lestrimelitta* specimens from the Yucatán Peninsula (Yuc=Yucatan State; QRoo=Quintana Roo State) and from Costa Rica (*Lestrimelitta danuncia*), with *Plebeia frontalis* as outgroup. Values show bootstrap branch support (500 replicates). The bar represents nucleotide sequence divergence.**

**Fig. S5. Detail of the propodeal spiracle, at least 4.6 times longer than broad (pointed by the yellow arrow), characteristic of *Lestrimelitta* species of the *ehrhardti* complex: left *L. niitkib*, right *Lestrimelitta sp.***

***
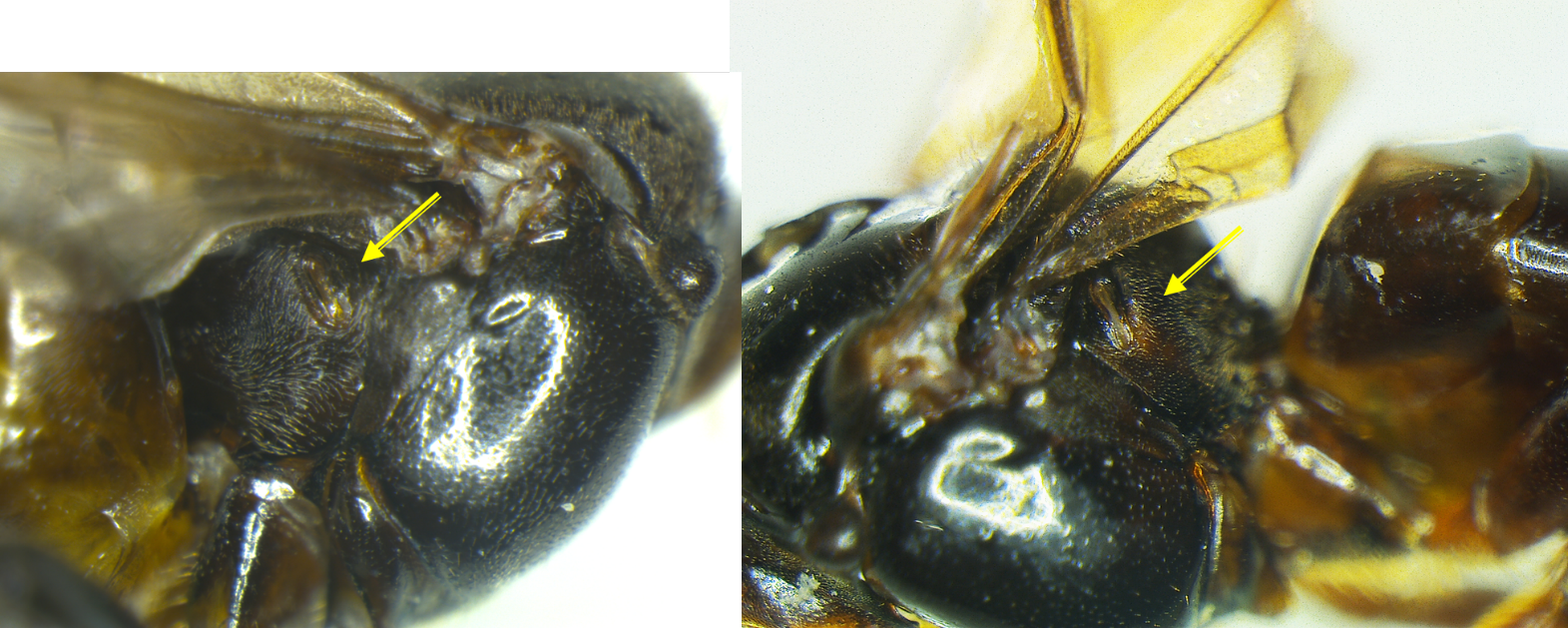
***
